# Principles of Human Physiology, with Their Chief Applications to Pathology, Hygiene, and Forensic Medicine

**Published:** 1847-12

**Authors:** 


ARTICLE XVII.

*Principles of Human Physiology, with their chief applications to Pathology, Hygiene, and Forensic Medicine.* By Wm. B. Carpenter, M. D., F. R. S., Fullerian Professor of Physiology in the Royal Institution of Great Britain, Corresponding Member of the American Philosophical Society, and of the National Institution of the United States, Lecturer on Physiology at the London Hospital, Medical School, etc. etc. Third American, from the last London Edition, with notes and additions by Meredith Clymer, M. D., Consulting Physician to the Philadelphia Hospital, late Professor of the Principles and Practice of Medicine and Clinical Medicine in the Franklin Medical College, Philadelphia, Fellow of the College of Physicians, etc. etc. etc., with three hundred and seventeen wood cuts and other illustrations. Philadelphia: Lea & Blanchard. 1847. (From the Publishers.)

Carpenter's Physiology is so well known through the medium of previous editions, that nothing need be said of the text. The additions by the editor, Dr. Clymer, naturally enhance the value of this edition, making it second to no work extant upon the subject of which it treats.

